# BMC Oral Health Reviewer Acknowledgment 2015

**DOI:** 10.1186/s12903-016-0178-z

**Published:** 2016-02-17

**Authors:** Elaine Zhang

**Affiliations:** BMC Oral Health, BioMed Central, 233 Spring Street, New York, NY 10013 USA

## Abstract

The editors of BMC Oral Health would like to thank all our reviewers who have contributed to the journal in Volume 15 (2015)

**Jenny Abanto**

Brazil

**Yoshihiro Abiko**

Japan

**Ahmet Hüseyin Acar**

Turkey

**Guy Adami**

USA

**Abiola Adeniyi**

Nigeria

**Joe Aduse-Opoku**

UK

**Himanshi Aggarwal**

India

**Hermann Agis**

Austria

**Bernardo Agostini**

Brazil

**Sang-Joon Ahn**

USA

**Jun Aida**

Japan

**Merve Akcay**

Turkey

**Mehmet Akin**

Turkey

**Gisele Caldas Alexandre**

Brazil

**Ahmad Aljafari**

UK

**Paul Allison**

Canada

**Vanessa Alves**

Brazil

**Manoharan Andiappan**

UK

**Flaviana Andrade**

Brazil

**Filippi Andreas**

Switzerland

**Oleh Andrukhov**

Austria

**Yulia Anopa**

UK

**Toshihiro Ansai**

Japan

**Raquel Antoniazzi**

Brazil

**Jose Leopoldo Ferreira Antunes**

Brazil

**Andreza Maria Aranha**

Brazil

**Thiago Ardenghi**

Brazil

**Rodrigo Arthur**

Brazil

**Paul Ashley**

UK

**Javier Ata-Ali**

Spain

**Sheyla Auad**

Brazil

**Joanna Baginska**

Poland

**Carolina Amalia Barcellos Silva**

Brazil

**Sergio Barros**

Brazil

**Renata Barroso De Carvalho**

Brazil

**Eraldo Batista**

Canada

**Alexandre Baumgarten**

Brazil

**Asli Baysal**

Turkey

**Sema Becerik**

Turkey

**Jonas Becktor**

Sweden

**Georgios Belibasakis**

Switzerland

**César Bergoli**

Brazil

**Gabriela Berti**

Brazil

**Kristina Bertl**

Austria

**Bishal Bhandari**

UK

**Vinodh Bhoopathi**

USA

**Silvio Diego Bianchi**

Italy

**Lars Bjørndal**

Denmark

**Fiona Blinkhorn**

Australia

**Paolo Boffano**

Italy

**Clarissa Bonifacio**

Netherlands

**Carolina Borges**

Brazil

**S. Aida Borges-Yanez**

Mexico

**Michael Bornstein**

Switzerland

**Ali Borzabadi-Farahani**

UK

**Nagihan Bostanci**

Switzerland

**Mariana Braga**

Brazil

**Carmela Bresolin**

Brazil

**Romina Brignardello-Petersen**

Canada

**Jonathan Broadbent**

New Zealand

**Josef Bruers**

Netherlands

**Nurcan Buduneli**

Turkey

**Bruno Bueno-Silva**

Brazil

**Yvonne Buischi**

USA

**Alexandre Bulgarelli**

Brazil

**Caroline Bulsara**

Australia

**Sandra Bussadori**

Brazil

**Azeez Butali**

USA

**John Butcher**

UK

**Michael Büttner**

Belgium

**William Buwembo**

Uganda

**Marilia Buzalaf**

Brazil

**Maria Grazia Cagetti**

Italy

**Rodrigo Cançado**

Brazil

**Peter Carlsson**

Sweden

**Thiago Carvalho**

Switzerland

**Luciano Casagrande**

Brazil

**Luiza Cassiano**

Brazil

**Paula Midori Castelo**

Brazil

**Paula Castelo**

Brazil

**Talita Castro**

Brazil

**Frank Catalanotto**

USA

**Ali Cekici**

Turkey

**Roger Keller Celeste**

Brazil

**Benjamin Chaffee**

USA

**Po-Chun Chang**

Taiwan

**Ava Chow**

Canada

**Bruno Chrcanovic**

Sweden

**Bradley Christian**

Australia

**Chun-Hung Chu**

Hong Kong

**Sasanka Chukkapalli**

USA

**Renata Cimoes**

Brazil

**Ayse Basak Cinar**

Denmark

**Georg Conrads**

Germany

**David Conway**

UK

**Marcos Britto Correa**

Brazil

**Marcos Corrêa**

Brazil

**Patrícia Corrêa-Faria**

Brazil

**Luciane Costa**

Brazil

**Yuri Costa**

Brazil

**Luis Cota**

Brazil

**Leonard Crocombe**

Australia

**David Crowe**

USA

**Carla Cugini**

USA

**Carolina Ortigosa Cunha**

Brazil

**Cristiane Da Mata**

Ireland

**Dorothea Dagassan**

Switzerland

**Anna Damanaki**

Germany

**Cees De Baat**

Netherlands

**Thiago De Marchi**

Brazil

**Patricia De Palma**

Sweden

**Harsha De Silva**

New Zealand

**Teun J. De Vries**

Netherlands

**Alberto Delbem**

Brazil

**Dong Deng**

Netherlands

**James Deschner**

Germany

**Matthias Dettmer**

Switzerland

**Ana Paula Dias Ribeiro**

Brazil

**Christina Diogo Löfgren**

Sweden

**Rafael Ditterich**

Brazil

**Kimon Divaris**

USA

**Ezgi Doğanay**

UK

**Ewa Dolinska**

Poland

**Sophie Doméjean**

France

**Mojtaba Dorri**

Iran, Islamic Republic Of

**Jennifer Doss**

Malaysia

**Poliana Duarte**

Brazil

**Joyce Duarte**

Brazil

**Denise Duijster**

Netherlands

**Walter Dukic**

Croatia

**Alexandrina Lizica Dumitrescu**

Norway

**Tom Dyer**

UK

**Kurt Ebeleseder**

Austria

**Sigrun Eick**

Switzerland

**Kim Ekstrand**

Denmark

**Tarek El-Bialy**

Canada

**Azza El-Housseiny**

Egypt

**Arjuna Ellepola**

Kuwait

**Bruno Emmanuelli**

Brazil

**Stefano Eramo**

Italy

**Gülfem Ergün**

Turkey

**Vanessa Euzebio Alves**

Brazil

**Wendell Evans**

Australia

**Megan Falsetta**

USA

**Ali Farahani**

UK

**Gisele Faria**

Brazil

**Nasim Fazel**

USA

**Georg Feichtinger**

UK

**Robert Fell**

Australia

**Giovana Fernandes**

Brazil

**Karin Fernandes**

Brazil

**Maurizio Ferrante**

Italy

**Patricia Ferrari**

Brazil

**Efigênia Ferreira**

Brazil

**Carlos Marcelo Figueredo**

Brazil

**John Firriolo**

USA

**Edgar-Arturo Flores-Larios**

Mexico

**Morenike Folayan**

Nigeria

**Lyndie Foster Page**

New Zealand

**Ashraf Fouad**

USA

**Deng Fu**

China

**Piotr Fudalej**

Switzerland

**Tsuyoshi Fujita**

Japan

**Peter Gaengler**

Germany

**Ana Gamboa**

UK

**Jinlong Gao**

Australia

**Iad Gharib**

UK

**Barry Gibson**

UK

**Fiona Gilchrist**

UK

**Thais Gimenez**

Brazil

**Nikolaos Gkantidis**

Switzerland

**Werner Goetz**

Germany

**Ali Golkari**

Iran, Islamic Republic Of

**Viviane Elisângela Gomes**

Brazil

**Lucio Gonçalves**

Brazil

**Raúl González-García**

Spain

**Morris Gordon**

UK

**Melissa Grant**

UK

**Ana Flavia Granville-Garcia**

Brazil

**Reinhard Gruber**

Austria

**Renata Guedes**

Brazil

**Pinar Gumus**

Turkey

**Orachad Gururatana**

Thailand

**Alex Nogueira Haas**

Brazil

**Sebastian Hahnel**

Germany

**Hassan Halawany**

Saudi Arabia

**Demetrios Halazonetis**

Greece

**Violet Haraszthy**

USA

**Melanie Hayes**

Australia

**Chuanglong He**

China

**Lisa Heaton**

USA

**Eva Hedman**

Sweden

**Rebecca Helmreich**

USA

**Min-Suk Heo**

Korea, South

**Bruno S Herera**

Brazil

**Daniela Hesse**

Brazil

**Nora Hiivala**

Finland

**Bruce Hirsch**

USA

**Ha Hoang**

Australia

**Kristin Hoeft**

USA

**Marinella Holzhausen**

Brazil

**Kiyonobu Honma**

USA

**Stephen Hsu**

Singapore

**Hsiao-Ling Huang**

Taiwan

**Michael Hülsmann**

Germany

**Haizal Hussaini**

New Zealand

**Koji Inagaki**

Japan

**Nicola Innes**

UK

**Andrei Ionescu**

Italy

**Kazuyuki Ishihara**

Japan

**Atsonori Isozaki**

Japan

**Kanade Ito**

Japan

**Masanori Iwasaki**

Japan

**Yuichi Izumi**

Japan

**Reinhilde Jacobs**

Belgium

**Joanna Janiszewska-Olszowska**

Poland

**Claude Jaquiery**

Switzerland

**Juliana Jardim**

Brazil

**Fawad Javed**

USA

**Newell Johnson**

Australia

**Birgitta Jönsson**

Norway

**Beuy Joob**

Thailand

**Horng-Heng Juang**

Taiwan

**Roger Junges**

Norway

**Luis Junquera**

Spain

**Jessica Kajfasz**

USA

**Thomas George Kallarakkal**

Malaysia

**Atsushi Kameyama**

Japan

**Ines Kapferer**

Austria

**Ertugrul Karatas**

Turkey

**Matthias Karl**

Germany

**Konstantinos Katsoulis**

Switzerland

**Mehmet Emin Kaval**

Turkey

**Katerina Kavvadia**

Greece

**Hakki Oguz Kazancioglu**

Turkey

**Moritz Kebschull**

Germany

**Ali Keles**

Turkey

**Muhammad Khalil Khan**

Saudi Arabia

**Kyeong-Seop Kim**

Korea, South

**Shiho Kino**

Japan

**Shiho Kino**

UK

**Atsuhiro Kinoshita**

Japan

**Mitsuo Kishi**

Japan

**Yuichi Kitasako**

Japan

**Marlise Klein**

Brazil

**Marlise Klein**

USA

**Cristiane Koga-Ito**

Brazil

**Klaus König**

Netherlands

**Dorota Kopycka-Kedzierawski**

USA

**Sudaduang Krisdapong**

Thailand

**Jeroen Kroon**

Australia

**Sebastian Kühl**

Switzerland

**Katherine Kula**

USA

**Wayne Kye**

USA

**Elena Labajo González**

Spain

**Giuseppina Laganà**

Italy

**Ratilal Lalloo**

Australia

**Jerónimo Lazos**

Argentina

**Soraya Leal**

Brazil

**Song Lee**

Canada

**Gillian Lee**

Hong Kong

**André Leite**

Brazil

**Claudio Leles**

Brazil

**Edina Lempel**

Hungary

**Tathiane Lenzi**

Brazil

**Roos Leroy**

Belgium

**Charlotte Lewis**

USA

**Ming-Yu Li**

China

**Lingling Li**

USA

**Sungwoo Lim**

USA

**Ramille Lima**

Brazil

**Huan-Cai Lin**

China

**Tülay Lindberg**

Sweden

**Ulrika Lindmark**

Sweden

**Giovanni Lodi**

Italy

**Carl Lombard**

South Africa

**Tommaso Lombardi**

Switzerland

**Peter Loomer**

USA

**Bruno Loos**

Netherlands

**Rodrigo Lopez**

Denmark

**Lucianne Maia**

Brazil

**Arash Mansourian**

Iran, Islamic Republic Of

**Medeiros Mara**

Mexico

**Rodrigo Mariño**

Australia

**Merete Markvart**

Denmark

**Zoe Marshman**

UK

**F.S. Martelli**

Italy

**Conchita Martin**

Spain

**Maria Carolina Martins Mussi**

Brazil

**Stephen Mason**

UK

**Manu Mathur**

India

**Yusuke Matsuyama**

Japan

**Juliana Mattos-Silveira**

Brazil

**Joanna May**

UK

**Mara Medeiros**

Mexico

**Noshir Mehta**

USA

**May Mei**

Hong Kong

**Peter Meisel**

Germany

**Mahtab Memarpour**

Iran, Islamic Republic Of

**Michel Messora**

Brazil

**Theodorus (Dirk) Mettes**

Netherlands

**Hendrik Meyer-Lückel**

Germany

**Fabio Mialhe**

Brazil

**Margaret Miller**

Australia

**Steve Miller**

USA

**Kyung-San Min**

Korea, South

**Richard Miron**

Canada

**Eduardo Moffa**

Brazil

**Osman Mohamed Osama Osman**

Switzerland

**Anelise Montagner**

Brazil

**Deborah Moore**

UK

**Rafael Moraes**

Brazil

**John Moran**

UK

**Carlos Moreira**

Brazil

**Annie Morgan**

UK

**Susan Morison**

UK

**Toshiya Morozumi**

Japan

**Gordon Morris**

UK

**Alireza Moshaverinia**

USA

**Jan Mulder**

Netherlands

**Heinz Müller**

Switzerland

**Wondemagegn Mulu**

Ethiopia

**Gonca Mumcu**

Turkey

**Brian Muzyka**

USA

**Rahul Naidu**

Trinidad And Tobago

**Rahul Nair**

Singapore

**Orawan Nammontri**

Thailand

**A Maya Nandkumar**

India

**Keiko Naruse**

Japan

**Gustavo Nascimento**

Brazil

**Declan Naughton**

UK

**Robert Nedelcu**

Sweden

**Tim Newton**

UK

**Dominique Niesten**

Netherlands

**Peter Nijkamp**

Netherlands

**Yoshikaki Nomura**

Japan

**Ryota Nomura**

Japan

**Hiraishi Noriko**

Japan

**Shimizu Noriyoshi**

Japan

**Ana Nunes**

Brazil

**Enihomo Obadan-Udoh**

USA

**Yorimasa Ogata**

Japan

**Taiji Ogawa**

Japan

**Takahiko Oho**

Japan

**Guilherme Oliveira**

Brazil

**Denise Oliveira**

Brazil

**Lucy O'Malley**

UK

**Anna-Lena Ostberg**

Sweden

**Kazuhisa Ouhara**

Japan

**Elizabeth Oziegbe**

Nigeria

**Damla Özsu**

Turkey

**V. Özgen Öztürk**

Turkey

**Keerthilatha Pai**

India

**Nikolaos Pandis**

Switzerland

**Gianpaolo Papaccio**

Italy

**Moschos Papadopoulos**

Greece

**George Papantonopoulos**

Greece

**Masoud Parirokh**

Iran

**Renato Parsekian Martins**

Brazil

**Shankargouda G. Patil**

India

**Antonio Carlos Pereira**

Brazil

**Luciano Pereira**

Brazil

**Karen Peres**

Australia

**Marco Peres**

Australia

**Juliano Pessan**

Brazil

**Ove Peters**

USA

**Elizabeth Philipone**

USA

**Lucas Pinheiro**

Brazil

**Ericka Pinheiro**

Brazil

**Chaiana Piovesan**

Brazil

**Fabiane Piva**

Brazil

**Antonella Polimeni**

Italy

**Deborah Polk**

USA

**Andre Priede**

Australia

**Laura Primo**

Brazil

**Sumant Puri**

USA

**Parth Purwar**

India

**Gert Jan Putten**

Netherlands

**Maryam Rabiei**

Iran

**Daniela Raggio**

Brazil

**Smrithi Rajendiran**

USA

**Karthikeyan Ramalingam**

India

**Joana Ramos-Jorge**

Brazil

**Cameron Randall**

USA

**Sarbin Ranjitkar**

Australia

**Amornrat Ratanasiri**

Thailand

**Nilantha Ratnayake**

Sri Lanka

**Daniel Reissmann**

Germany

**Torsten W. Remmerbach**

Germany

**Daniela Rios**

Brazil

**Agneta Robertson**

Sweden

**Peter Robinson**

UK

**Rachel Rocha**

Brazil

**Laura Roesch**

Mexico

**Matthias Roggendorf**

Germany

**Ellen Rogo**

USA

**Marina Roscoe**

Brazil

**Martin Rosentritt**

Germany

**Gabriele Rossini**

Italy

**Rodnei Dennis Rossoni**

USA

**Patrick Rouxel**

UK

**R Gary Rozier**

USA

**Ingrid Rozylo-Kalinowska**

Poland

**Jianping Ruan**

China

**Thiago Saads Carvalho**

Switzerland

**Omer Sagsoz**

Turkey

**Philipp Sahrmann**

Switzerland

**Meenu Saini**

USA

**Atsushi Saito**

Japan

**Christian Salazar**

USA

**Katayoun Salem**

Iran

**Humam Saltaji**

Canada

**Vidya Sankar**

USA

**Mauro Pedrine Santamaria**

Brazil

**Oseas Santos Junior**

Brazil

**Terukazu Sanui**

Japan

**Yukihiro Sato**

Japan

**Anurag Satpathy**

India

**Edgar Schäfer**

Germany

**Benoit Schaller**

Switzerland

**Rob Mh Schaub**

Netherlands

**Andrea Maria Schmidt-Westhausen**

Germany

**Anja Schmitt**

Switzerland

**Dirk Schulze**

Germany

**Joel Schwartz**

USA

**Uwe Schwarze**

Austria

**Don Schwass**

New Zealand

**Helmut Schweikl**

Germany

**Falk Schwendicke**

Germany

**David Scott**

USA

**Hidenobu Senpuku**

Japan

**Benedict Seo**

New Zealand

**Junia Serra-Negra**

Brazil

**Salvatore Settineri**

Italy

**Rabia Gonul Sezer**

Turkey

**Siddharth Shanbhag**

India

**Koki Shioya**

Japan

**Bernd W. Sigusch**

Germany

**Emmanuel Silva**

Brazil

**Lucas Hian Silva**

Brazil

**Cleverson Silva**

Brazil

**Chelsia Sim**

Singapore

**Alyne Simoes**

Brazil

**Ankur Singh**

Australia

**Ripudaman Singh**

India

**Shenuka Singh**

South Africa

**Carla Sipert**

Brazil

**Gustavo Sivieri-Araujo**

Brazil

**Anna Skalniak**

Poland

**Marit S Skeie**

Norway

**Anna Skurska**

Poland

**Linda Slack-Smith**

Australia

**Adriana Soares**

Brazil

**Francesca Soldani**

UK

**Mikael Sonesson**

Sweden

**Gianrico Spagnuolo**

Italy

**Heiko Spallek**

USA

**Alexandra Stähli**

Switzerland

**Joao Paulo Steffens**

Brazil

**Patricia-Anca Steinmassl**

Austria

**Emelie Stenberg**

Sweden

**Simon Stone**

UK

**Stefan-Ioan Stratul**

Romania

**Juliana Stuginski-Barbosa**

Brazil

**Rüstem Kemal Sübay**

Turkey

**Karin Sunnegårdh-Grönberg**

Sweden

**Nao Suzuki**

Japan

**Koichi Tabeta**

Japan

**Hiroyuki Tada**

Japan

**Santosh Kumar Tadakamadla**

India

**Anwar Tappuni**

UK

**Tamara Tedesco**

Brazil

**Tellervo Tervonen**

Finland

**Thomas Thurnheer**

Switzerland

**Tamanna Tiwari**

USA

**Mimmi Tolvanen**

Finland

**Fernanda Tomazoni**

Brazil

**Utsana Tonmukayakul**

Australia

**Huseyin Topcuoglu**

Turkey

**Hüseyin Sinan Topçuoglu**

Turkey

**Lidia Tordiglione**

Italy

**Markus Troeltzsch**

Germany

**Simone Tuchtenhagen**

Brazil

**Anne Bjørg Tveit**

Norway

**Sergio Uribe**

Chile

**Vinay V Kumar**

India

**Diego Valentim**

Brazil

**Monique Van Der Veen**

Netherlands

**Denise Van Diermen**

Netherlands

**Fabiana Vargas-Ferreira**

Brazil

**Carlalberta Verna**

Switzerland

**Christopher Vernazza**

UK

**Lance Vernon**

USA

**Mario Vettore**

UK

**Stella Vieira**

Brazil

**Alexandre Rezende Vieira**

USA

**Rui Miguel Pinheiro Vitorino**

Portugal

**Rodrigo Vivan**

Brazil

**Ana Vukovic**

Serbia

**John Walters**

USA

**Zilu Wang**

China

**Shuai Wang**

Hong Kong

**Qian Wang**

USA

**Saman Warnakulasuriya**

UK

**Michael Wefelmeier**

Germany

**Florian J. Wegehaupt**

Switzerland

**Lam Wendy**

Hong Kong

**Annette Wiegand**

Germany

**Tove Irene Wigen**

Norway

**Leslie Will**

USA

**Brita Willershausen**

Germany

**Peter Windisch**

Hungary

**Sarah Wong**

Hong Kong

**Raymond Wong**

Singapore

**Kyung Mi Woo**

Korea, South

**Devina Worsley**

UK

**Jibieke Wulaerhan**

China

**Alidianne Xavier**

Brazil

**Masaru Yamaguchi**

Japan

**Tatsuo Yamamoto**

Japan

**Duygu Yaman**

Turkey

**Yan Yang**

China

**Ghaeth Yassen**

USA

**Ibrahim Yaylali**

Turkey

**Ping Ye**

Australia

**Veerasamy Yengopal**

South Africa

**Anthony Yeo**

Australia

**Akihiro Yoshida**

Japan

**Rodrigue Yossa Nouaga**

Canada

**Rosnah Binti Zain**

Malaysia

**Carlos Zaror**

Chile

**Piero Antonio Zecca**

Italy

**Florian Zeman**

Germany

**Lin Zeng**

USA

**Wenjian Zhang**

USA

**Lai-Ping Zhong**

China

**Zhi Zhou**

China

**Xiaofei Zhu**

China

**Thomas Ziebart**

Germany

**Stefan Zimmer**

Germany

**Brigitte Zimmerli**

Switzerland

**Argyro Zougli**

Germany

